# The Effect of Fluid Overload on Attributable Morbidity after Cardiac Surgery: A Retrospective Study

**DOI:** 10.1155/2020/4836862

**Published:** 2020-12-04

**Authors:** Vildan Koc, Laura Delmas Benito, Eldert de With, E. Christiaan Boerma

**Affiliations:** Department of Intensive Care, Medical Center Leeuwarden, Leeuwarden, Netherlands

## Abstract

**Introduction:**

Although the detrimental effects of excessive perioperative fluid administration are generally well established, data in the setting of cardiac surgery remain less robust.

**Methods:**

In this retrospective single-center observational study, the total fluid balance in the first 12 hours during and after surgery was evaluated. Primary endpoint was the relationship between total fluid balance and the incidence of prolonged mechanical ventilation. For this purpose, data were divided into quartiles (Q); prolonged mechanical ventilation and prolonged length of stay (LOS) in the ICU were defined as Q4. Secondary endpoints were prolonged LOS in the ICU, incidence of acute kidney injury (AKI; defined as a 1.5-fold increase in serum creatinine during, relative to baseline), and ICU mortality.

**Results:**

In a 3-year period, 748 patients were included. In a univariate analysis, the median duration of mechanical ventilation was 2.9 h [2.4–3.7] in Q1 of the fluid balance and increased significantly to 4.7 h [3.1–9.2] in Q4 of the fluid balance (*p* < 0.001). In addition, patients in Q4 of the fluid balance had a significantly longer LOS in the ICU, as well as a higher incidence of AKI and ICU mortality. In a multivariate analysis, Q4 of the fluid balance was independently associated with prolonged mechanical ventilation (OR 4.9, CI 2.9–8.4, *p* < 0.001) and prolonged LOS in the ICU (OR 11.3 CI 6.1–20,9, *p* < 0.001), but not with the incidence of AKI or ICU mortality.

**Conclusions:**

Perioperative fluid administration in cardiac surgery patients was independently associated with prolonged mechanical ventilation and prolonged LOS in the ICU.

## 1. Introduction

In critical care, there is growing concern about the potential detrimental effects of fluid administration [1]. Although fluid therapy remains the foundation of shock resuscitation, fluid overload is associated with longer mechanical ventilation, renal failure, and even mortality [1, 2]. This is well demonstrated in the critically ill patients, especially in the patients with sepsis and acute lung injury. Several studies have demonstrated improved patient outcome with conservative fluid therapy [2–8]. However, in the setting of cardiac surgery, this association is less well established. Indeed, some studies observed a clear association between fluid overload and adverse outcome in the specific setting of congenital heart surgery [9, 10]. Others reported a correlation between fluid balance and acute kidney injury (AKI) in the general cardiac surgery population, with the lowest incidence in patients with zero fluid balance [11]. However, data on additional important outcome variables, including mechanical ventilation, remain to be established.

In this study, we aimed to determine whether the fluid balance is independently associated with the time to extubation following cardiac surgery.

## 2. Materials and Methods

### 2.1. Study Design and Patient Selection

A single-center retrospective observational study was performed, involving all cardiovascular surgery patients (≥18 years) admitted to the ICU of the Medical Center Leeuwarden between August 2014 and May 2017. Emergency cases and preoperative renal replacement therapy dependent cases were not excluded from the primary analyses. However, two patients on preoperative renal replacement therapy were excluded from the secondary endpoint analyses on kidney function. A local ethical committee (Regionale Toetsingscomissie Patientgebonden Onderzoek, Leeuwarden, the Netherlands) waived the need for informed consent according to applicable laws (nWMO 2017, 236). The preliminary data of this study have been published before in an abstract form during the 38^th^ International Symposium on Intensive care and Emergency Medicine [12].

### 2.2. Data Collection

Several hospital electronic databases and information systems (Metavision and Mediscore) were used to extract data, and all data were recorded anonymously. This included demographics (age and sex), comorbidities, cardiac status at time of operation, pre- and postoperative left ventricular ejection fraction (LVEF), and the type of procedure. Risk scores, including the Acute Physiology and Chronic Health Evaluation (APACHE III) score, European System for Cardiac Operative Risk Evaluation (EuroSCORE), and New York Heart Association (NYHA) class III or IV), as well as fluid balance and the use of inotropes, were recorded.

### 2.3. Postoperative Details

All postoperative patients were admitted to the ICU while still on mechanical ventilation. The settings of mechanical ventilation were standardized according to local protocols. Extubation was performed after the first 2 hours of ICU admission, in case of hemodynamic stability and absence of complications (bleeding, infarction). The main reason for fluid administration was hypotension (mean arterial pressure <65 mmHg) in combination with a low central venous oxygen saturation (SvO_2_ <60%).

### 2.4. Endpoints

Primary endpoint was the relationship between the fluid balance and the incidence of prolonged mechanical ventilation. For this purpose, data were divided into quartiles (Q); prolonged mechanical ventilation and prolonged length of stay (LOS) in ICU were defined as Q4. Secondary endpoints were prolonged LOS in the ICU, incidence of AKI, and ICU mortality. Additionally, we explored the relationship between fluid balance and (rise of) central venous pressure (CVP). AKI was diagnosed according to the Kidney Disease Improving Global Outcome (KDIGO) criteria as a 1.5-fold increase in serum creatinine (sCr) relative to preoperative sCr [11, 13]. Mean CVP was measured during the first 2 hours of ICU admission to make groups comparable by minimizing the influence of mechanical ventilation on this outcome.

### 2.5. Statistical Analysis

The Statistical Package for Social Science (SPSS version 24 SPSS inc., Chicago, IL, USA) was used. Data are represented as median [interquartile range, IQR], unless stated otherwise. The data were tested for normal distribution using the Kolmogorov–Smirnov test. In case of abnormal distribution, an applicable nonparametric test (Kruskal–Wallis) was used to detect differences between groups. If significant differences were detected, a Mann–Whitney *U* test was used to compare each group with one another. All statistics were two-tailed, and a *p* value <0.05 was considered statistically significant.

Due to the lack of specific knowledge on the distribution of fluid balance, a proper power analysis was impossible. A sample size of 750 patients was deemed sufficient in accordance with the previous literature [14].

A multivariate linear regression analysis was performed using the backward Likelihood Ratio (LR) method. Continuous variables were transformed into binominal variables (e.g., prolonged vs. nonprolonged mechanical ventilation), using the 75^th^ percentiles as cutoff values. Baseline characteristics with a *p* value <0.25 in the univariate analysis were included in the model.

Ideally, before conducting the multivariate linear regression analysis, correction for missing data should be performed. However, this was the case in less than 2% of the patient population, excluding 10 out of a total of 750 patients. Therefore, it was decided not to perform a procedure for multiple imputations.

## 3. Results

A total of 750 patients were included in this study. Two patients were excluded afterwards due to incorrect reason for admission. The mean age was 68 ± 10 years, and 71.3% were male. The total fluid balance ranged from −2.6 to 11.0 L. The baseline characteristics are summarized in Table 1. Differences between quartiles of fluid balance were observed in gender, history of neurological dysfunction, additive EuroSCORE, and APACHE III. In detail, patients with the highest fluid balance were younger, had less often a history of neurological dysfunction, and had a higher EuroSCORE and APACHE III score. No differences were found with respect to cardiac status and preoperative LVEF.

Perioperative characteristics are summarized in Table 2. Significant differences between quartiles of fluid balance were observed in all fields, except for the use of dobutamine.

### 3.1. Primary Endpoint

**Figure 1:**
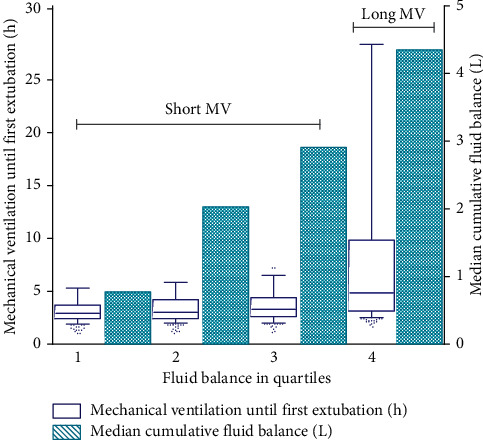
Fluid balance and duration of mechanical ventilation.

In a univariate analysis, patients with the highest total fluid balance (Q4) had significantly longer duration of mechanical ventilation compared to patients in the other quartiles of fluid balance (Figure 1). The median duration of mechanical ventilation was 2.9 h [2.4–3.7] in Q1 of the fluid balance and increased significantly to 4.7 h [3.1–9.2] in Q4 of the fluid balance (*p* < 0.001, Table 3). The percentage of patients with a prolonged duration of mechanical ventilation was 12% in Q1 of the fluid balance as compared to 47% in Q4 of the fluid balance (*p* < 0.001). In a multivariate analysis, the fluid balance remained an independent risk factor for prolonged mechanical ventilation (Table 4).

### 3.2. Secondary Endpoints

**Figure 2:**
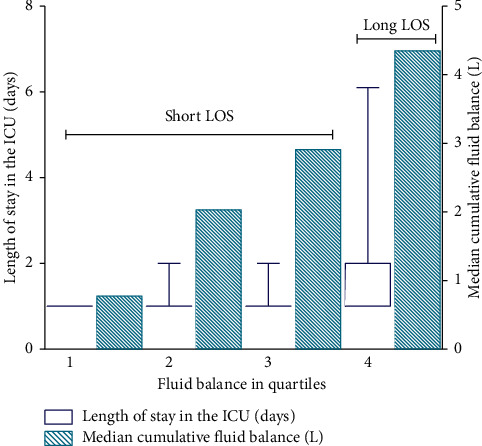
Fluid balance and length of stay (LOS) in the ICU.

In a univariate analysis, patients with the highest total fluid balance (Q4) had a significantly longer LOS in the ICU (Figure 2), as well as the highest incidence of AKI and ICU mortality. Although the median duration of LOS in the ICU, expressed as absolute values, was similar between groups, the difference was statistically significant due to the rise in incidence of outliers. The percentage of patients with prolonged LOS in the ICU was 9% in Q1 of the fluid balance as compared to 37% in Q4 of the fluid balance (*p* < 0.001). The incidence of AKI was 0.5% in Q1 of the fluid balance and increased significantly to 4.8% in Q4 of the fluid balance (*p* < 0.007). ICU mortality was 0.5% in Q1 of the fluid balance and increased significantly to 3.2% in Q4 of the fluid balance (*p* < 0.025). Although absolute mortality was lower in Q3 as compared to Q1 and Q2, this was not statistically significant. Furthermore, a moderately but significantly higher CVP was observed in Q4. After multivariate analysis, Q4 of the total fluid balance remained an independent risk factor for prolonged mechanical ventilation and LOS in the ICU, but not for the incidence of AKI or ICU mortality (Table 4).

## 4. Discussion

In this study, there was an independent association between fluid administration and the incidence of prolonged mechanical ventilation. Although the absolute difference in median duration of mechanical ventilation between Q1 and Q4 is “only” 1.8 hours, Figure 1 clearly shows the clinical burden created by the substantial number of outliers. The clinical relevance was further illustrated by the fact that the Q4 of the fluid balance over the first 12 hours was also independently associated with prolonged mechanical ventilation and LOS in the ICU.

A potential explanation is the extravasation of fluid into the extracellular compartment, driven by increased hydrostatic pressure. This extravasation may result in pulmonary edema and decreased pulmonary function and, finally, prolong the duration of mechanical ventilation [6, 15–18]. Additional contributing factors include hemodilution and the release of proinflammatory mediators, with subsequent increased capillary permeability and organ dysfunction, reflected by extended hospitalization and ICU mortality [14, 19].

The observed detrimental effects of fluid overload on LOS in the ICU in this study are consistent with previous studies [11, 14, 20]. Several authors reported a significant and independent effect of fluid overload on combined events (including death) and LOS in the ICU in cardiosurgical patients [11, 21]. Although not confirmed in a multivariate model, the observed increase in the incidence of AKI between the quartiles of fluid balance is in line with recent studies [19, 22]. Of note, although CVP was significantly higher in our data, the absolute difference in CVP was only 1 mmHg, unlikely to be detected in the clinical setting. Others linked fluid balance to the use of renal replacement therapy [9, 10]. The effect of fluid overload on the duration of mechanical ventilation is well established in pediatric congenital heart surgery, but to our knowledge, we are the first to evaluate this in adults. In line with this study, previous studies have also found that the maximum cumulative fluid balance was strongly correlated with the duration of mechanical ventilation [10, 23]. Moreover, multiple studies show that not only fluid overload is detrimental but that a conservative strategy of fluid management yields a better outcome [4, 8, 24]. Although the vast majority of current literature is based on noncardiac surgery in critically ill patients, our study illustrates that fluid overload is independently associated with morbidity in the cardiac surgery setting as well.

## 5. Limitations

The present study had several limitations. Due to the retrospective and single-centered design, the inevitable evaluation over time carries the potential of unrecognized biases, and the potential for generalization to other settings is limited. Additionally, severity of illness, hemodynamic monitoring techniques and endpoints, fluid management protocols, and postoperative complications may be possible confounders and may not be fully accounted for in this retrospective analysis. Although the study lacks an a priori sample size calculation, post hoc sample size calculation reveals that as little as 5 patients per group would be sufficient to detect a significant difference in the primary endpoint between Q1 and Q4.

Ideally, a large randomized controlled trial of a fluid restricted versus liberal fluid strategy would be needed to definitively determine the cause-effect relationship of fluid overload on attributable to morbidity after cardiac surgery.

## 6. Conclusions

Perioperative fluid administration in cardiac surgery patients was independently associated with prolonged mechanical ventilation and prolonged LOS in the ICU.

## Figures and Tables

**Table 1:** Baseline characteristics.

	Q1 *n* = 186	Q2 *n* = 188	Q3 *n* = 187	Q4 *n* = 187	*p* value
Fluid balance ranges (L)	−2.6–1.5	1.5–2.4	2.4–3.5	3.5–11.0	

Demographics					
Age (years)	68.0 [61–76]	67.5 [61–75]	68.0 [62–75]	70.0 [63–76]	0.315
Male (%)	78.5	72.3	72.2	62.0	0.005^*∗*^^a,b,c^
BMI (kg/m^2^)	26.8 [24.5–29.7]	26.6 [24.4–29.9]	26.8 [24.6–30.5]	26.9 [24.8–29.3]	0.870
Diabetes mellitus (%)	20.4	24.5	25.7	23.0	0.662
Hypertension (%)	39.8	51.1	49.2	52.9	0.054
Peripheral vessel disease (%)	13.4	15.4	14.4	12.8	0.896
TIA/CVA (%)	9.1	13.8	12.8	11.8	0.537
Neurological dysfunction (%)	3.2	7.4	3.7	1.1	0.014^*∗*^^b^
COPD (%)	12.4	16.5	19.3	11.8	0.137
Preoperative sCR (*μ*mol/L)	84.0 [74–98]	82.0 [71–99]	81.0 [70–98]	88.0 [74–103]	0.214

Cardiac status (%)					
NYHA class III or IV	64.7	52.7	54.5	54.0	0.096
Recent myocardial infarction	24.2	18.1	18.2	16.6	0.253
Previous cardiac surgery	24.2	22.9	21.9	20.3	0.836

Preoperative LVEF (%)					
Good	74.2	69.1	78.1	75.4	0.160
Moderate	19.9	19.1	17.1	18.2	
Poor	5.9	11.7	4.8	6.4	

Risk scores					
Additive EuroSCORE	5 [3–7]	5 [3–8]	4 [3–7]	6 [4–8]	0.007^*∗*^^a,b,c^
APACHE III	49 [40–57]	45 [36–57]	46 [38–57]	49 [42–62]	0.016^*∗*^^b,c^

Data are expressed as median [interquartile range] or as percentage. BMI, body mass index; TIA, transient ischemic accident; CVA, cerebral vascular accident; COPD, chronic obstructive pulmonary disease; sCR; serum creatinine; NYHA, New York Heart Association; LVEF, left ventricular ejection fraction; LOS, length of stay; EuroSCORE, European System for Cardiac Operative Risk Evaluation; APACHE, Acute Physiology and Chronic Health Evaluation. ^*∗*^Groups are significantly different when *p* < 0.05. ^a^: significant difference between Q1 and Q4; ^b^: significant difference between Q2 and Q4; ^c^: significant difference between Q3 and Q4.

**Table 2:** Perioperative characteristics.

	Q1 *n* = 186	Q2 *n* = 188	Q3 *n* = 187	Q4 *n* = 187	*p* value
Type of procedure (%)					
CABG	71.0	53.7	63.1	34.2	<0.001^*∗*^^a,c,d,e,f^
AVR	8.1	16.0	10.7	20.9	
CABG + AVR	2.7	6.9	11.8	27.3	
MVR	7.0	5.9	5.3	4.3	
Other	11.3	17.6	9.1	13.4	

Intraoperative characteristics					
ACC (min)	59.0 [36–83]	53.5 [32–77]	55.0 [39–75]	70.0 [46–109]	<0.001^*∗*^^c,e,f^
ECC (min)	85.0 [56–116]	82.0 [48–111]	81.0 [58–107]	105.0 [68–148]	<0.001^*∗*^^c,e,f^

Postoperative LVEF (%)					
Good	73.1	73.4	84.5	78.1	0.038^*∗*^^b,d^
Moderate	21.0	18.1	13.9	16.6	
Poor	5.9	8.5	1.6	5.3	

Use of noradrenaline (%)	12.9	23.4	18.2	32.1	<0.001^*∗*^^a,c,f^
Use of dobutamine (%)	10.8	14.9	11.2	18.7	0.095
Postoperative sCR (*μ*mol/L)	88 [73–104]	86 [72–102]	86 [69–104]	93 [73–116]	0.045^*∗*^^e,f^

Data are expressed as median [interquartile range] or as percentage. CABG, coronary artery bypass grafting; AVR, aortic valve repair; MVR, mitral valve repair; ACC, aortic cross clamp; ECC, extracorporal circulation; sCR, serum creatinine. ^*∗*^Groups are significantly different when *p* < 0.05. ^a^: significant difference between Q1 and Q2; ^b^: significant difference between Q1 and Q3; ^c^: significant difference between Q1 and Q4; ^d^: significant difference between Q2 and Q3; ^e^: significant difference between Q2 and Q4; ^f^: significant difference between Q3 and Q4.

**Table 3:** Univariate analyses.

	Q1 n = 186	Q2 n = 188	Q3 n = 187	Q4 n = 187	*p* value
Duration of mechanical ventilation (hours)	2.9 [2.4–3.7]	3.0 [2.4–4.2]	3.3 [2.6–4.4]	4.7 [3.1–9.2]	<0.001^*∗*^^a,b,c,d^
LOS in the ICU (days)	1.0 [1.0–1.0]	1.0 [1.0–1.0]	1.0 [1.0–1.0]	1.0 [1.0–2.0]	<0.001^*∗*^^b,c,d^
CVP	9 [6–11]	9 [7–11]	9 [7–11]	10 [8–12]	0.003^*∗*^^b,c,d^
Incidence of AKI (%)	0.5	1.1	1.1	4.8	0.007^*∗*^^b,c,d^
ICU mortality (%)	0.5	1.1	0.0	3.2	0.025^*∗*^^d^

Data are expressed as median [interquartile range] or as percentage. LOS, length of stay; CVP, central venous pressure; AKI, acute kidney injury; ICU, intensive care unit. ^a^: significant difference between Q1 and Q3; ^b^: significant difference between Q1 and Q4; ^c^: significant difference between Q2 and Q4; ^d^: significant difference between Q3 and Q4.

**Table 4:** Multivariate analyses.

Multivariate analysis	OR (95% CI)	*p* value
Prolonged mechanical ventilation (>4.9 hours)		
Quartiles of total fluid balance (Q)		
Q2	1.449 (0.813–2.585)	0.209
Q3	1.690 (0.956–2.987)	0.071
Q4	4.938 (2.901–8.403)	<0.001
Additive EuroSCORE	1.090 (1.027–1.157)	0.005
ACC (min)	1.006 (1.002–1.010)	0.003
Use of noradrenaline	1.616 (1.016–2.571)	0.043

Prolonged LOS in ICU (≥2 days)		
Quartiles of total fluid balance (Q)		
Q2	0.955 (0.460–1.982)	0.901
Q3	3.284 (1.728–6.243)	<0.001
Q4	11.31 (6.12–20.96)	<0.001
Additive EuroSCORE	1.172 (1.097–1.252)	<0.001
ACC (min)	1.009 (1.005–1.014)	<0.001
Use of noradrenaline	2.247 (1.403–3.599)	0.001

LOS, length of stay; AKI, acute kidney injury; EuroSCORE, European System for Cardiac Operative Risk Evaluation; ACC, aortic cross clamp; ICU, intensive care unit. The first quartile (Q1) of total fluid balance served as the reference value.

## Data Availability

The data are available from the corresponding author with the permission of the Medical Center Leeuwarden. The restrictions apply to the availability of these data, which were used under license for this study.

## Abbreviations

ACC: Aortic cross clamp
AKI: Acute kidney injury
APACHE: Acute Physiology and Chronic Health Evaluation
AVR: Aortic valve repair
BMI: Body mass index
CABG: Coronary artery bypass grafting
COPD: Chronic obstructive pulmonary disease
CVA: Cerebral vascular accident
CVP: Central venous pressure
ECC: Extracorporal circulation
EuroSCORE: European System for Cardiac Operative Risk Evaluation
ICU: Intensive care unit
KDIGO: Kidney disease improving global outcome
LOS: Length of stay
LR: Likelihood ratio
LVEF: Left ventricular ejection fraction
MVR: Mitral valve repair
NYHA: New York Heart Association
sCR: Serum creatinine
SPSS: Statistical package for social science
TIA: Transient ischemic accident.
